# Mind-body approaches for reducing the need for post-operative opioids: Evidence and opportunities

**Published:** 2022

**Authors:** Bethany D. Pester, Robert R. Edwards, Marc O. Martel, Christopher J. Gilligan, Samantha M. Meints

**Affiliations:** 1 Department of Anesthesiology and Pain Medicine, Brigham and Women’s Hospital, Harvard Medical School, Boston, USA; 2 Faculty of Dentistry and Department of Anesthesiology, McGill University, Montreal, Canada

## Introduction

While opioids remain our most potent analgesics in the management of pain, the many potential harms of prescription opioids have become increasingly clear. Despite the analgesic benefits for people with acute and chronic pain [1], opioid therapy (especially long-term opioid treatment) can result in significant problems such as opioid misuse, the development of opioid use disorder, and overdose. Some authors report that up to 20-30% of patients in primary and tertiary care settings who are maintained on long-term opioid therapy misuse opioids (i.e., use them in a manner other than how the opioids are prescribed) [2,3]. Misuse of opioids can cause or exacerbate additional health problems in people with chronic pain [1,4], and in fact, roughly 10% of patients prescribed long-term opioid therapy may develop an opioid use disorder (OUD), although prevalence estimates vary between studies depending on differences in methodology and operational definitions [3]. Surgery is a common point of exposure to opioids for many individuals, and recent data suggest that substantial percentages of opioid-naive patients will go on to long-term opioid use following a surgery (e.g., approximately 6% in one large national survey [5]). With over 50 million surgeries performed in the US each year [6], the peri- and post-operative period represents an important window of opioid-related risk for many Americans.

In a prior review [7], we described patient characteristics associated with elevated risk for opioid use problems. For instance, patients presenting with concomitant psychotropic medication use and/or a lifetime history of a mental disorder(s) are at greater risk of opioid misuse and OUD [8–11]. Psychosocial characteristics that have been consistently linked with opioid-related problems include: anxiety [8,12–17], trauma history and/or post-traumatic stress disorder [17–20], depressive symptoms (particularly anhedonia or loss of pleasure) [17,21–27], high levels of negative affect [8,12,13,23,24,28–32], pain catastrophizing or a negative cognitive and emotional response to actual or anticipated pain (particularly rumination) [28,33–37], personality traits such as impulsivity and sensation-seeking [38,39], borderline and antisocial personality disorders [8,39], sleep disturbances [9,35,40–42], and deficits in pain coping skills [15,43–48]. Psychological factors appear to be more predictive of opioid misuse and OUD than pain-related factors such as the type of pain condition experienced by a patient or even a patient’s reported pain intensity [12–14,22,28,33,34,49,50], which might be expected to serve as a very strong predictor. Psychological characteristics, such as high levels of depression, anxiety, and insomnia, are also shown to predict long-term opioid use after surgery, among other predictors such as pre-surgical opioid use (which is invariably among the strongest determinants of post-operative opioid use) and a history of substance use disorder(s) [51–53]. This is a potentially quite important but at times under-appreciated point: many of the same factors that confer risk for long-term opioid use in the setting of chronic pain also serve as risk factors for heavy use, misuse, and persistent use of opioids after a surgical procedure. Given that psychosocial factors appear to be predominant drivers of opioid use problems across settings, it seems sensible to target those factors in order to improve opioid-related outcomes. It is not surprising, then, that psychological interventions for chronic pain have subsequently emerged to reduce risks and potential harms associated with opioid therapy.

Psychological or mind-body interventions have historically been included as part of multidisciplinary treatment for chronic pain and are designed to reduce pain intensity, psychological distress, and pain-related disability. Mind-body interventions (MBIs) confer their benefits via multiple mechanisms, some of which are physiological in nature. For example, MBIs have been shown to positively affect heart rate variability (HRV) in chronic pain patients, which is important as chronic pain has been strongly linked to low HRV mediated by reduced parasympathetic activity [54]. While initially designed to improve outcomes such as pain severity and pain-related disability, the goals of these interventions have expanded over time to include reduction or prevention of opioid use problems among patients with chronic pain. Recent studies suggest that MBIs may reduce the need for and misuse of opioids by remediating dysfunctions in reward and autonomic systems [55,56]. Mind-body approaches for managing chronic pain and opioid misuse often incorporate elements from cognitive behavioral therapy (CBT; e.g., education about chronic pain and opioid misuse, coping skills to deal with pain and opioid cravings, activity pacing, stress management, sleep hygiene) and acceptance and commitment therapy (ACT; e.g., values-based behavioral activation, mindfulness, cognitive defusion, acceptance). Recently published trials of mind-body treatments often utilize interventions that blend components from multiple therapeutic orientations, including CBT, ACT, Pain Neuroscience Education, and Emotional Awareness and Expression Therapy (for example, see [57]). The efficacy of such psychological interventions in treating chronic pain and opioid use problems has been well-established (for review, see [7]).

## MBIs in Perioperative Settings

Recent work has revealed the potential of mind-body treatments (originally studied as interventions to alleviate pre-existing persistent pain) to critically reduce pain and opioid use after surgery. Surgery can precipitate both chronic pain and long-term opioid use [58,59], with approximately 30% of patients developing chronic, postsurgical pain [60]. Currently, pharmacological pain management is standard peri- and post-operative practice [6]. However, given the risks associated with opioids and sedatives, there is an undeniable need for empirically-supported non-pharmacological interventions to prevent long-term sequelae of post-operative pain and opioid use. MBIs, which have been effectively integrated into the treatment of patients with established chronic pain, have been adapted to prevent the transition from acute postsurgical pain, to optimize pharmacological management of chronic pain and reduce the risk of prolonged opioid use after surgery.

Recent studies of MBIs delivered during the perioperative period show promise for reducing pain and opioid use after surgery. Hadlandsmyth and colleagues [61] conducted a pilot randomized controlled trial (RCT) of the Perioperative Pain Self-Management (PePS) intervention; a 4-session, telephone-based CBT approach, adapted to target acute postsurgical pain management for veterans. Sessions focused on behavioral pain management skills such as relaxation training, cognitive restructuring and use of coping thoughts, and goal-setting. Findings suggest that this brief structured pre- and post-surgical psychological pain self-management intervention may lower rates of moderate-to-severe surgical site pain and opioid use following surgery. At 3-months post-surgery, only 7% of patients who received the PePS intervention reported moderate-to-severe pain compared to 26% who received standard care, and only 2% of PePS participants were taking opioids compared to 15% of controls. Qualitative analyses indicated that PePS participants benefitted from learning to think differently about pain, find ways to take their mind off pain, and use pain self-management skills to reduce their reliance on medications and/or increase time between doses. Though concise, feasible, and scalable (particularly given the telephone-based delivery) to implement, this brief intervention did require some flexibility in terms of timing and structure of sessions.

Another pilot study [62] similarly utilized a remote, flexible, and low-cost format (i.e., automated mobile phone messaging) to deliver a CBT-based intervention to musculoskeletal tumor patients scheduled for outpatient surgery. They found that patients who received the virtual CBT intervention (i.e., daily text messages giving post-operative guidance and encouragement and inquiring about pain and opioid use) showed a gradual reduction in pain intensity and opioid utilization over the first two post-operative weeks, with nearly all patients reporting that they were no longer using opioids by the end of the second post-operative week. Experimental participants had significantly lower opioid utilization rates than controls, who did not receive virtual CBT messaging. Patients were generally accepting of the post-operative communication platform, with the majority reporting feeling more connected to their team and preferring to conduct post-operative communication via text messaging, which represents a highly convenient, patient-friendly mode of interaction that is likely worth considering in future MBI studies.

A larger, three-arm RCT (N=118) tested the effects of two mindfulness approaches for patients undergoing total joint arthroplasty: mindfulness of breath (MoB) and mindfulness of pain (MoP) [63]. The two approaches mainly differed in the target of attention. MoB trained participants in focused attention on the breath, metacognitive monitoring, and acceptance of automatic thoughts, negative emotions, and body sensations. MoP, which called for focused attention on both pain and neutral/pleasant sensations, provided interoceptive exposure to pain, helped patients to separate physical sensations from automatic emotional reactions and pain appraisals, and shift attention to nonpainful sensations. MoB and MoP were both delivered in a single, 20-minute session approximately 3 weeks before surgery and were compared with a standard behaviorally-focused psychoeducation intervention delivered in the same format. Of the patients who received either mindfulness approach, only 2% were taking opioids one month after surgery, compared to 40% in the psychoeducation comparison condition. MoB appeared most effective at decreasing immediate preoperative pain, whereas MoP resulted in the least amount of postoperative pain and pain interference at 1-month follow-up.

Finally, a recent RCT by Flowers et al. [64] deviated from the standard cognitive-behavioral and mindfulness-based approaches by using classical conditioning to pair opioid analgesics (an unconditioned stimulus) with placebo pills (a conditioned stimulus) in an open-label placebo paradigm (i.e., participants receiving placebo pills are informed that placebos are known to activate natural endogenous mechanisms that alleviate pain) to elicit pain reduction in response to placebos alone. Among spinal fusion patients, compared to treatment as usual, the conditioned open-label placebo treatment was associated with approximately 50% less daily opioid consumption following discharge from the hospital. Moreover, the conditioned open-label placebo group showed an earlier discontinuation from opioids after surgery, as well as lower levels of worst pain during the post-operative period. The benefits of this intervention were most pronounced in younger patients, women, and those with higher preoperative pain severity; historically these have been the surgical candidates at the highest risk for severe and long-term post-surgical pain, suggesting that this non-pharmacologic intervention is maximally effective in those who are most in need of such treatments.

## Summary

In sum, brief psychological interventions delivered in the perioperative period show promise in preventing both chronic postsurgical pain and prolonged opioid use for many surgical patients. Psychological interventions for pain have historically been used to treat established chronic pain conditions and opioid use problems, or as one-time interventions to improve acute pain in a medical setting (e.g., relaxation and distraction techniques for children undergoing painful procedures). Recent work has elucidated novel ways in which MBIs can be tailored to the perioperative period to prevent persistent pain and long-term opioid use after surgery in both opioid-naive patients and patients on pre-operative opioid regimens. Such MBIs show tremendous promise as supplemental treatments in the context of multi-modal management of peri- and post-operative pain. Many of these treatments demonstrate both short- and long-term benefits (i.e., reduced pain, reduced need for opioids), with minimal potential for harms, or adverse effects; moreover, they can be applied as adjunctive treatments to existing regimens, as they do not interfere with (and may even offer synergies with) pharmacologic treatments, physical treatments such as physical therapy, etc. The brevity and low cost of mind-body interventions make them feasible to implement across settings and patient populations. It is noteworthy that many of these treatments require only a small number of sessions/visits, and most are amenable to remote delivery, making them potentially highly scalable.

Historical impediments to psychological intervention, in general, include cost, logistical difficulties such as requirements for multiple in-person patient visits, and lack of access to providers with expertise in delivering these interventions. Increasingly, these barriers are becoming less salient as MBIs are available by remote delivery and covered by third-party payers. The delivery of psychological intervention during the perioperative period, however, introduces new challenges given certain logistical constraints such as timing of surgery. MBIs will need to be designed to maximize their flexibility in terms of format and delivery; already, a number of researchers have been creatively experimenting with: (1) the structure and timing of sessions (e.g., single sessions [63]; pre- and post-surgical timing of the treatments), (2) remote delivery platforms such as mobile phone messaging, and (3) innovative approaches such as conditioned placebos and focused mindfulness strategies.

Overall, these approaches appear to hold great promise for improving pain- and opioid-related outcomes following the tens of millions of surgical procedures that are performed every year in the U.S. alone. The risks associated with long-term opioid therapy cannot be ignored and the large (and growing) daily toll of opioid deaths is a clear call for ongoing work in this area. At this point, numerous hospital systems have begun implementing Enhanced Recovery After Surgery (ERAS) protocols to optimize hydration, nutrition, and pain control, leading to faster, safer, and more comfortable recovery from surgery. In general, most ERAS programs do not currently include MBIs in their multimodal treatment packages. Adding MBIs to existing ERAS protocols may present a valuable opportunity to further enhance the efficacy of a second generation of ERAS programs. Given the sizable (and growing) body of evidence that MBIs can improve acute outcomes in the peri- and post-operative space, and that MBIs show substantial long-term effects in preventing persistent pain and long-term need for opioids after surgery, such treatments appear to offer highly favorable risk-benefit profiles. Simultaneously, larger RCTs with flexible delivery formats and long-term assessment are needed to test and refine perioperative mind-body approaches to prevent the development of chronic pain and opioid use problems after surgery.
